# ﻿A new species of *Rhyacophila* Pictet, 1834 (Trichoptera, Rhyacophilidae) from Corsica with the genomic characterization of the holotype

**DOI:** 10.3897/zookeys.1218.132275

**Published:** 2024-11-22

**Authors:** Ernesto Rázuri-Gonzales, Wolfram Graf, Jacqueline Heckenhauer, Julio V. Schneider, Steffen U. Pauls

**Affiliations:** 1 Senckenberg Research Institute and Natural History Museum Frankfurt, Frankfurt, Germany Senckenberg Research Institute and Natural History Museum Frankfurt Frankfurt Germany; 2 Institute of Hydrobiology and Aquatic Ecosystem Management (IHG), University of Natural Resources and Life Sciences, Vienna, Austria University of Natural Resources and Life Sciences Vienna Austria; 3 LOEWE Centre for Translational Biodiversity Genomics (LOEWE-TBG), Frankfurt, Germany LOEWE Centre for Translational Biodiversity Genomics (LOEWE-TBG) Frankfurt Germany; 4 Institute of Insect Biotechnology, Justus-Liebig University, Gießen, Germany Justus-Liebig University Gießen Germany

**Keywords:** Caddisflies, holotype genomics, mitogenome, taxonomy

## Abstract

We describe a new species in the *Rhyacophilatristis* group, *Rhyacophilalignumvallis* Graf & Rázuri-Gonzales, **sp. nov.**, from the island of Corsica (France) based on a single male specimen. In addition to the morphological differences between the new species and the most similar species in the group, we also provide a phylogenetic tree based on the mitochondrial cytochrome *c* oxidase subunit I (mtCOI), including sequences from 16 out of the 28 currently recognized species in the group. These data, together with conspecific larval sequences, support the status of the new species and shed light on an additional potential new species near *Rhyacophilapubescens*. Using a low-cost next-generation sequencing approach, we generated the mito- and draft nuclear genome assembly of the holotype of *R.lignumvallis***sp. nov.** as well as that of *R.tsurakiana*. This genetic data represents an important additional characterization to the description of morphological features and is valuable for future investigations, such as population or phylogenomic studies.

## ﻿Introduction

The genus *Rhyacophila*Pictet 1834, with 814 extant and 30 fossil species, is the largest caddisfly genus in the world (Valladolid et al. 2023). These caddisflies are primarily distributed in the northern hemisphere, but they also occur in temperate and tropical India and southeastern Asia (Holzenthal et al. 2007). Given the sheer size of the genus, many species groups have been proposed based on the morphological characteristics of the larvae (Döhler 1950) and the male genitalia (Ross 1956; Schmid 1970). However, the groups and subgenera proposed by different authors do not correspond to each other. For example, some of the larval characters for the subgenera of Döhler are found in several of the groups of Schmid. Phylogenetic studies of different groups proposed by Schmid (1970) seem to showcase the overall utility of his system, even if rearrangements are sometimes needed (Coppa et al. 2012).

One of the groups proposed by Schmid is the *Rhyacophilatristis* group in the *R.invaria* branch. It is mainly characterized by a large segment IX without an apical dorsal lobe, a simple and oblique segment X, with fairly large anal sclerites, which can be joined or partially fused to each other and fused to segment X, very large phallotheca with a simple or complex dorsal arm, a simple chitinous aedeagus, simple lobe-like parameres, and lacking a ventral lobe, among other characteristics (Schmid 1970). Currently, the species group contains 28 species in two subgroups: the *tristis* and the *pubescens* subgroups (Schmid 1970, Suppl. material 1). The *tristis* subgroup is further characterized by the complex shape of the dorsal arm of the phallotheca and the presence of fairly large parameres fused to the aedeagus. The *pubescens* subgroup, on the other hand, has a simpler dorsal arm of the phallotheca and elongated free parameres. Species of the *R.tristis* species group (i.e., both subgroups) generally occur in headwaters to medium-sized, fast-flowing streams at middle elevations throughout Europe (excluding the British Isles, Northern Europe, and Russia) and Western Asia (i.e., Turkey and Iran) (Schmid 1970; Coppa et al. 2012; Suppl. material 1).

Only two species in the *R.tristis* group were previously known on the island of Corsica (France): *R.pubescens* Pictet, 1834 and *R.tristis* Pictet, 1834. Both species were initially recorded by Mosely (1930, 1932). More recently, Engelhardt (2009) assessed the phylogenetic relationships in the *R.tristis* group and the phylogeography of *R.pubescens* throughout its distributional range. Engelhardt showed that the larval specimens of *R.pubescens* from the island were significantly divergent from all the other populations. Moreover, the Corsican specimens formed a highly supported clade, separate from all other *R.pubescens* (Engelhardt et al. 2011).

In the present paper, we confirm the genetic and morphological distinctness of adult males of the Corsican lineage to represent a new species and describe it as *Rhyacophilalignumvallis* sp. nov. in the *pubescens* subgroup of the *R.tristis* group. Additionally, we present an annotated draft genome from the holotype and its complete mitogenome, adding valuable genetic information to the holotype description. Finally, we also include a draft genome and the mitogenome for a second species in the *Rhyacophilatristis* species group, *R.tsurakiana* Malicky, 1984 from Albania. We hope these genomic resources stimulate research on this group of insects, especially since their diversity is high in this area and they seem to be restricted to specific mountain ranges, as suggested by Oláh et al. (2022).

## ﻿Material and methods

### ﻿Specimen collection

The specimen was swept from the riparian vegetation using a hand net.

### ﻿DNA extraction, library preparation, and whole genome sequencing

A pair of legs from the holotype of *R.lignumvallis* sp. nov. were removed, and the tissue was incubated overnight in 60 µl TNES lysis buffer (100 mM Tris-HCl, 25 mM NaCl, 10 mM EDTA, 1% SDS) and 8 µl Proteinase K (20 mg/ml). For DNA binding and cleanup, 75 µl custom speed-bead suspension was added (Sera-Mag SpeedBeads Carboxylate, hydrophobic, Cytiva; see Rohland and Reich 2012), incubated for 15 min on a rotating shaker, and the beads were washed twice with 75% ethanol after the supernatant had been removed and discarded. The DNA was eluted in 1X TE.

DNA sequences were generated for the cytochrome *c* oxidase subunit I barcoding region (mtCOI, 658 bp) using primers LCO1490-L and HCO2198-L (Nelson et al. 2007). Polymerase chain reactions (PCR) were run on a Mastercycler Pro S (Eppendorf, Hamburg, Germany) in reactions containing 1X MyTaq Reaction Buffer, 0.4 µM of each forward and reverse primer, 0.5 U MyTaq DNA Polymerase, 1 µl DNA and nuclease-free water to fill up to a total volume of 10 µl. Reaction conditions were 1 min at 95 °C for initial denaturation followed by 35 cycles of 20 s at 95 °C (denaturation), 30 s at 45 °C (annealing), and 30 s at 72 °C (extension). The reaction ended with a final extension for 5 min at 72 °C. PCR products were visualized on agarose gels and purified using a modified ExoSAP protocol with Exonuclease I (20U/µl) and Fast AP Themosensitive Alkaline Phosphatase (1U/µl; both ThermoFisher Scientific, Vilnius, Lithuania). DNA sequences were generated at the Laboratory Centre of the Senckenberg Biodiversity and Climate Research Centre using a 3730XL DNA Analyzer (Applied Biosystems).

Genomic DNA was taken from the above DNA isolates, quantified using a Qubit 4.0 fluorometer with a 1x dsDNA HS Assay Kit (ThermoFisher Scientific, Waltham, USA), and sheared to a target fragment size of 350 bp using a Bioruptor Pico (Diagenode, Seraing, Belgium). Genomic libraries were prepared from 27.4 ng sheared gDNA using the NEBNext Ultra II DNA Library Preparation Kit for Illumina (New England Biolabs, Ipswich, MA, USA), following the manufacturer’s manual. Adapters were diluted 1:10 as recommended for low input libraries, and size selection was omitted due to the low DNA amount. A dual indexing PCR was run for 7 cycles on a Mastercycler (Eppendorf, Germany) using NEBNext Multiplex Oligos for Illumina (Dual Index Primers Set 1; New England Biolabs, Ipswich, MA, USA). After cleanup, library integrity was verified on a 2200 TapeStation with a High Sensitivity D1000 Tape (Agilent, Santa Clara, CA, USA), and shipped for 150 bp paired-end sequencing (ordering 30 Gbp output) on a partial lane of an Illumina NovaSeq 6000 platform (San Diego, CA) at Novogene (Cambridge, UK).

Raw reads are deposited in the National Center for Biotechnology Information’s Sequence Read Archive (NCBI SRA) under the accession number SRR22799047 under Bioproject PRJNA899095.

### ﻿DNA barcoding and phylogenetic analysis

The final mtCOI alignment included 71 sequences: 68 sequences from 16 species and 2 morphospecies in the *Rhyacophilatristis* group. *Rhyacophilaitalica* Moretti, 1981, *Himalopsychekuldschensis* (Ulmer, 1927), and *H.triloba* (Hwang, 1958) were included as outgroups. All sequences were generated for this manuscript, except *R.bosnica* Schmid, 1970 (MK211322), *H.kuldschensis* (KX143534), and *H.triloba* (KX295339), which were retrieved from GenBank. The barcode region is 658 bp in length. However, some of our sequences were incomplete, and their lengths were between 576 and 658 bp. Therefore, the final alignment was completed with Ns on both ends, reaching 3.69% of missing data. Sequence specimen data and GenBank accession numbers are summarized in Table 1.

**Table 1.** Sequence specimen data, with GenBank accession numbers, of the studied *Rhyacophila* species and the outgroups *Himalopsychekuldschensis* (Ulmer, 1927) and *H.triloba* (Hwang, 1958).

Species	Country*	Locality	Latitude, Longitude	Accession No.
*Himalopsychekuldschensis*	KG	Kalay Makhmud valley between Or-Mazan-Suu and Ala Malden	39.683, 70.8833	KX143534
*Himalopsychetriloba*	CN	Sichuan, near Jiuzhaigou	30.45, 102.50	KX295339
*R.akutila*	BG	Prava Marica stream at Zavracica mountain hut	42.16789, 23.64139	PP515197
*R.aquitanica*	FR	Ruisseau de Chousse, upper tributary; between Arrette & La Pierre Saint-Martin	43.00757, -0.73572	PP515198
*R.aquitanica*	FR	Ruisseau de Chousse, upper tributary; between Arrette & La Pierre Saint-Martin	43.00757, -0.73572	PP515199
*R.aquitanica*	FR	Ruisseau de Chousse, upper tributary; between Arrette & La Pierre Saint-Martin	43.00757, -0.73572	PP515200
*R.aquitanica*	FR	Ruisseau de Chousse, upper tributary; between Arrette & La Pierre Saint-Martin	43.00757, -0.73572	PP515201
*R.aquitanica*	ES	tributaries to the Barranco de Urdiceto, above Embalse de Urdiceto	42.67832, -0.2772	PP515202
*R.bosnica*	BA	Vareš municipality, Rajčevački stream	–	MK211322
*R.carpathica*	RO	Galeş Lake	45.38650, 22.90914	PP515204
*R.carpathica*	RO	Galeş Lake	45.38650, 22.90914	PP515205
*R.carpathica*	RO	Galeş Lake	45.38650, 22.90914	PP515206
*R.carpathica*	RO	Galeş Lake	45.38650, 22.90914	PP515207
*R.carpathica*	RO	Caraş-Severin, Iauna Mare stream	45.51636, 22.59017	PP515208
*R.carpathica*	RO	Caraş-Severin, Poiana Mărului	45.39583, 22.53422	PP515209
*R.cibinensis*	RO	unnamed stream near Păltiniş	45.63878, 23.92540	PP515210
*R.cibinensis*	RO	unnamed stream near Păltiniş	45.63878, 23.92540	PP515211
*R.cibinensis*	RO	unnamed stream near Păltiniş	45.63878, 23.92540	PP515212
*R.cibinensis*	RO	Lotru river	45.38, 23.62	PP515213
*R.italica*	IT	Purello	43.32, 12.77	PP515214
*R.lignumvallis* sp. nov.	FR	Corsica, Tributary to the Tavignano	42.25639, 9.20583	PP515216
*R.lignumvallis* sp. nov.	FR	Corsica, Tributary to the Tavignano	42.25639, 9.20583	PP515217
*R.lignumvallis* sp. nov.	FR	Corsica, Tributary to the Tavignano	42.25639, 9.20583	PP515218
*R.lignumvallis* sp. nov.	FR	Corsica, bridge over the river Vecchio near the confluence with the river Tavignano	42.2275, 9.24306	PP515215
*R.margaritae*	BG	Lower left tributary to Zavodna, above Ribaritsa village and below Vezhen peak	42.76, 24.37	PP515219
*R.margaritae*	BG	Lower left tributary to Zavodna, above Ribaritsa village and below Vezhen peak	42.76, 24.37	PP515220
*R.margaritae*	BG	Lower left tributary to Zavodna, above Ribaritsa village and below Vezhen peak	42.76, 24.37	PP515221
*R.obtusa*	BG	Zavodna river, upstream of the confluence with the Beli Vit at the Ribaritsa village	42.812, 24.371	PP515222
*R.obtusa*	BG	Zavodna river, upstream of the confluence with the Beli Vit at the Ribaritsa village	42.791, 24.377	PP515223
*R.orghidani*	RO	right-side inflow of Leşu artificial lake	46.80981, 22.58948	PP515224
*R.orghidani*	RO	right-side inflow of Leşu artificial lake	46.80981, 22.58948	PP515225
*R.orghidani*	RO	Băişoara	46.53287, 23.28078	PP515226
*R.orghidani*	RO	Băişoara	46.53287, 23.28078	PP515227
*R.pirinica*	BG	24.5 km NNW from Gotse Delchev	41.63156, 23.44628	PP515228
*R.pirinica*	BG	24.5 km NNW from Gotse Delchev	41.63156, 23.44628	PP515229
*R.pirinica*	BG	24.5 km NNW from Gotse Delchev	41.63156, 23.44628	PP515230
*R.producta*	AT	Nockberge	46.85, 13.76	PP515231
*R.producta*	AT	Nockberge	46.85, 13.76	PP515232
*R.pubescens*	CH	La Motte above Ocourt	47.35, 7.06	PP515233
*R.pubescens*	CH	La Motte above Ocourt	47.35, 7.06	PP515234
*R.pubescens*	FR	Ravin de Chambiéres	43.93278, 6.63694	PP515235
*R.pubescens*	FR	Ravin de Chambiéres	43.93278, 6.63694	PP515236
*R.pubescens*	FR	La Condamine-Châtelard	44.451, 6.741	PP515237
*R.pubescens*	FR	La Condamine-Châtelard	44.451, 6.741	PP515238
*R.pubescens*	FR	La Condamine-Châtelard	44.451, 6.741	PP515239
*R.pubescens*	IT	Tributary of Fiume Tescio	43.09722, 12.67556	PP515240
*R.pubescens*	IT	Tributary of Fiume Tescio	43.09722, 12.67556	PP515241
*R.pubescens*	IT	Tributary of Fiume Tescio	43.09722, 12.67556	PP515242
*R.pubescens*	IT	Nameless brook near Rezzo	44.02583, 7.86667	PP515243
*R.pubescens*	IT	Valle di Pietra	44.07722, 7.80639	PP515244
*R.pubescens*	IT	Valle di Pietra	44.07722, 7.80639	PP515245
*R.sarplana*	AL	Tropojë, open stream on Mt. Callumit, above town	42.49862, 20.12443	PP515203
*Rhyacophila* sp., *tristis* grp.	AT	Carinthia, Gail river at Kötschach-Mauthen town	46.67, 12.98	PP515255
*Rhyacophila* sp., *tristis* grp.	IT	Lombardia, Valle del Ferro	45.77277, 9.98996	PP515256
*Rhyacophila* sp., *tristis* grp.	IT	Trentino-Alto Adige/Südtirol, Camposilvano	45.75988, 11.14189	PP515257
*Rhyacophila* sp., *tristis* grp.	FR	Ruisseau de Chousse, upper tributary; between Arrette & La Pierre Saint-Martin	43.00757, -0.73572	PP515258
*R.trescavicensis*	ME	Ali-pašini springs	42.54706, 19.83240	PP515246
*R.trescavicensis*	ME	Ali-pašini springs	42.54706, 19.83240	PP515247
*R.trescavicensis*	ME	Ali-pašini springs	42.54706, 19.83240	PP515248
*R.trescavicensis*	ME	Ali-pašini springs	42.54706, 19.83240	PP515249
*R.tristis*	RO	Hunedoara, Câmpu lui Neag	45.30227, 22.97388	PP515250
*R.tristis*	RO	Hunedoara, Câmpu lui Neag	45.30227, 22.97388	PP515251
*R.tristis*	RO	Covasna, Comandău	45.81488, 26.32934	PP515252
*R.tristis*	RO	Harghita, Voşlăbeni	46.6815, 25.6738	PP515253
*R.tristis*	RO	Vâlcea, Voineasa, Lotru river	45.463, 23.62	PP515254
*R.tsurakiana*	AL	river Shushica at the village of Brataj	40.26622, 19.67198	PP515259
*R.vranitzensis*	BA	Sljeme	43.9403, 18.5122	PP515260
*R.vranitzensis*	BA	Sljeme	43.9403, 18.5122	PP515261
*R.vranitzensis*	BA	Sljeme	43.9403, 18.5122	PP515262
*R.vranitzensis*	BA	Skakavac waterfall	43.94238, 18.44196	PP515263
*R.vranitzensis*	BA	Skakavac waterfall	43.94238, 18.44196	PP515265

* Country codes in the ISO 3166-1 alpha-2 standard

The maximum likelihood tree was produced using IQ-TREE v.2.1.3 (Minh et al. 2020), using the command *iqtree2 -s RhyacophilaLignumvalleMS_658bp.fasta -B 1000 -bnni -alrt 1000 --prefix RhyacophilaLignumvalleMS_658bp*. The TIM2+F+I+G4 nucleotide substitution model was selected using ModelFinder (Kalyaanamoorthy et al. 2017). Statistical support for the tree topology was assessed with the ultrafast bootstrap approximation (UFboot) (Hoang et al. 2017). Clades with UFboot ≥ 95% are considered well-supported. Additionally, each bootstrap tree was optimized with a hill-climbing nearest neighbor interchange (NNI) search (flag -bnni in the command above) based on the corresponding bootstrap alignment to prevent overestimating UFboot branch support values, as recommended by Hoang et al. (2017).

The consensus tree was visualized and edited in TreeViewer v.2.2.0 (Bianchini and Sánchez-Baracaldo 2024). Additional aesthetic edits were made in Adobe Illustrator CS6.

### ﻿Holotype mitogenome and nuclear genome assembly

After quality control with FastQC v.0.11.9 (Andrews 2019), raw reads were trimmed for low-quality regions, adapter sequences, and overrepresented *k-mers* using autotrim.pl v.0.6.1 (Waldvogel et al. 2018) and Trimmomatic v.0.39 (Bolger et al. 2014) with the adapter_all.fa of Trimmomatic and the following settings ILLUMINACLIP:2:30:10:8:true, SLIDINGWINDOW:4:20, MINLEN:50, and TOPHRED33. Unpaired reads were discarded and paired reads were checked for contamination using Kraken v.2.0.9 (Wood and Salzberg 2014).

Genome size was estimated using a method based on *k-mer* distribution. For this, *k-mers* were counted with JELLYFISH v.2.3.0 (Marçais and Kingsford 2011) using *jellyfish count -C -s 1000000000 -F 2* and a k-mer length of 21 based on the raw sequence reads. A histogram of *k-mer* frequencies was created with *jellyfish histo* and used for analysis with the online web tool GenomeScope v.2.0 (Ranallo-Benavidez et al. 2020) using the following parameters: *k-mer* length = 21, ploidy = 2, max *k-mer* coverage = 10000.

The mitochondrial genomes were first assembled with the raw reads using NOVOplasty v.4.2 (Dierckxsens et al. 2016) using the following parameters: type = mito, genome range = 12000–22000, *k-mer* = 33, max memory = 100, read length = 150, insert size = 300, platform = illumina, paired = PE, insert size auto = yes. The partial sequence of the cytochrome *c* oxidase subunit I (COX1) gene of *Rhyacophilafasciata* Hagen, 1859 (MT559357.1) was used as seed input. All other parameters were kept as default. In addition, we used a second mitogenome assembler MitoZ v.2.3 (Meng et al. 2019). For this purpose, the raw data was subsampled to 10,000,000 reads using seqk and then used as input for *MitoZ assemble* with the following parameters: genetic_code 5, clade Arthropoda, fastq_read_length 150, insert_size 300, run_mode 2, filter_taxa_method 1, requiring_taxa ‘Arthropoda’. Annotation of tRNAs, rRNAs, and protein-coding genes was done for the best mitogenome assembly of each species with MitoZ v.2.3 using the module annotate with genetic_code 5 and clade Arthropoda. Both mitogenome assemblies were aligned to the complete mitogenome of *R.quadrifida* Sun & Yang, 1995 (OL678049.1) and *R.kando* Schmid, 1970 (OL678048.1) with MAFFT in Geneious Prime v.2022.1.1 (Biomatters Ltd.) to set the correct start position and manually curate the control-region. The mitochondrial genome assembly was deposited in GenBank under the accession OQ984043.

Nuclear genome assembly was conducted in Spades v.3.14.1 (Bankevich et al. 2012) with the default settings. Scaffolds smaller than 500 bp and those with blast hits to the mitochondrial genome assembly were filtered out. Assembly statistics were calculated with Quast v.5.0.2 (Gurevich et al. 2013) and completeness was assessed via screening for single-copy orthologs with BUSCO v.5.2.2 (Manni et al. 2021) using the endopterygota_odb10 dataset. As an additional quality control, trimmed reads were mapped back to the assembly with bwa-mem v.0.7.17-r1188 (Li 2013) with parameters -a -c 10000, and the back-mapping rate was calculated with qualimap v.2.2.1 (Okonechnikov et al. 2015). To check for potential contamination, taxon-annotated GC-coverage (TAGC) plots were generated with BlobTools v.1.1.1 (Laetsch and Blaxter 2017). For this purpose, the bam file resulting from the back-mapping analysis was converted to a blobtools readable cov file with *blobtools map2cov*. Taxonomic assignment for BlobTools was conducted with blastn v.2.10.0+ (Camacho et al. 2009) using -task megablast and -e-value 1e-25. The blobDB was created and plotted with the cov file and blast hits. The nuclear draft genome assembly was deposited in GenBank under accession JAPMAE000000000. The DNA barcode region was extracted from the genome assembly and aligned to the traditionally sequenced mtCOI sequences and showed 100% identity to the larvae included by Engelhardt et al. (2011).

Genomic methods were identical for *R.tsurakiana* (see Suppl. material 3).

### ﻿Morphological examination

The holotype specimen was prepared and examined following standard methods for ethanol-preserved material (Blahnik and Holzenthal 2004; Blahnik et al. 2007). Forewing length was measured from base to apex with a microscale (BioQuip Products, Rancho Dominguez, California, USA).

The abdomen was removed from the specimen, soaked in 85% lactic acid, and heated to 99 °C for 60 min to dissolve internal soft tissues. The macerated tissues were then flushed out of the abdomen with a syringe. The holotype was examined on an Olympus SZX10 stereomicroscope, and pencil sketches of the genitalia were prepared using a Leitz Dialux 20 compound microscope outfitted with a drawing tube. Pencil sketches were scanned and placed in an Adobe Illustrator CS6 document as a template for vector illustrations. Morphological terminology follows Schmid (1970) for the male genitalia, Holzenthal et al. (2007) for wing venation, and Ivanov (1990) for setal warts.

## ﻿Results

### ﻿Phylogenetic analysis

After collapsing clades with less than 70% bootstrap support, the species in the *Rhyacophilatristis* group were placed in a polytomy, and sister to *R.italica* (Fig. 2). The first clade of the polytomy includes *R.pubescens*, *R.tsurakiana*, and the new species. The holotype specimen (marked with an asterisk in Fig. 2) was included in a highly supported clade (100% bootstrap support) with three *Rhyacophila* larvae from Corsica. Based solely on larval identification, these were originally considered to be *R.pubescens* (Engelhardt 2009; Engelhardt et al. 2011). It now seems clear that these are larvae of *R.lignumvallis* sp. nov.

**Figure 1.**
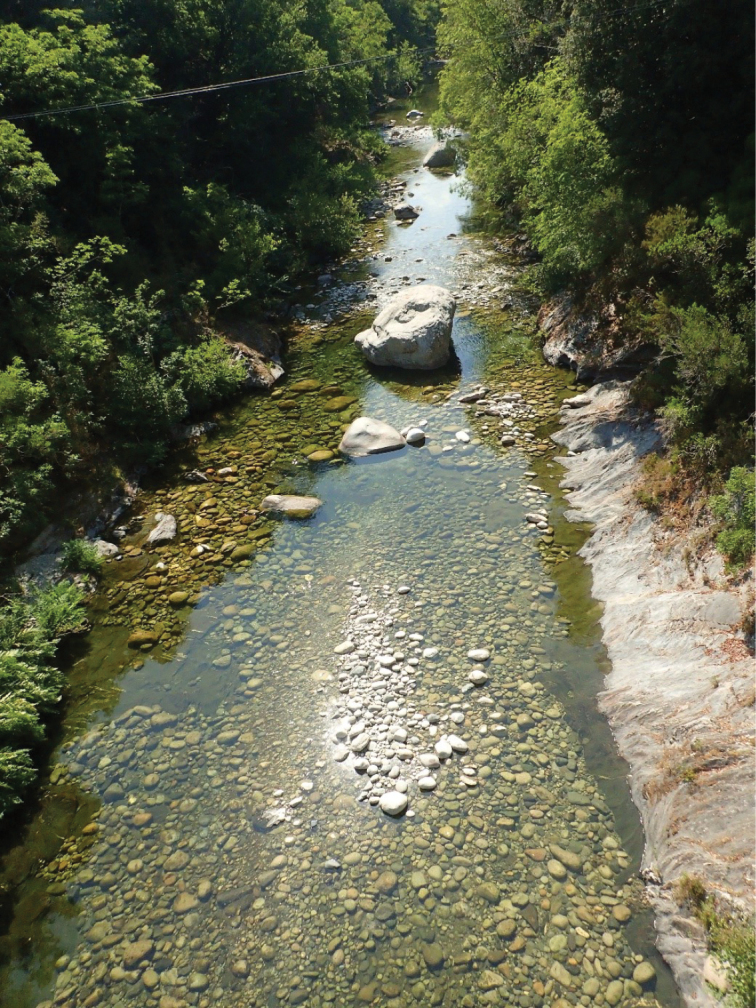
Type locality of *Rhyacophilalignumvallis* sp. nov. on the island of Corsica (France).

**Figure 2.**
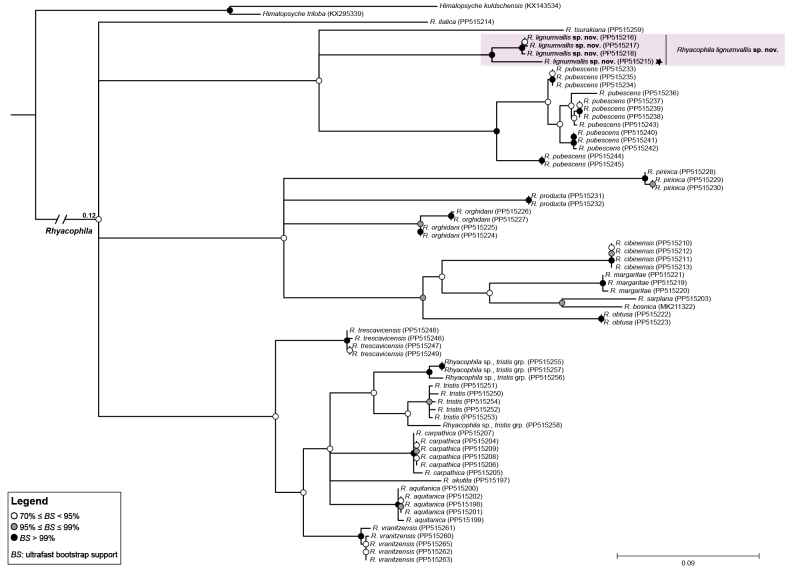
Phylogenetic relationships in the *Rhyacophilatristis* group based on the mtCOI barcode region and estimated by maximum likelihood, as implemented in IQ-TREE v.2.1.3 (Minh et al. 2020). Nodal support was calculated using the ultrafast bootstrap (UFboot) approximation (Hoang et al. 2017); nodes with UFboot values greater than 95% are considered well supported. Branches with support values of less than 70% were collapsed. Branch lengths are measured in nucleotide substitutions per site (see scale bar for reference). The branch leading to *Rhyacophila* was shortened (total branch length was 0.12 substitutions per site). Numbers in parentheses correspond to GenBank accession numbers. The holotype and the Corsican larvae are shaded.

The second clade in the polytomy includes several species from the *tristis* subgroup (*R.orghidani* Botosaneanu, 1952, *R.cibinensis* Botosaneanu & Marinkovic-Gospodnetic, 1967, *R.margaritae* Kumanski, 1998, *R.bosnica*, and *R.obtusa* Klapalek, 1894), and two species from the *pubescens* subgroup (*R.pirinica* Kumanski, 1980 and *R.producta* McLachlan, 1879). These specimens were collected in Albania, Austria, Bulgaria, and Romania. The third clade in the polytomy exclusively includes species from the *tristis* subgroup (*R.aquitanica* McLachlan, 1879, *R.carpathica* Botosaneanu, 1995, *R.trescavicensis* Botosaneanu, 1960, *R.tristis* Pictet, 1834, *R.vranitzensis* Botosaneanu & Marinkovic-Gospodnetic, 1967, and *Rhyacophila* sp.). These specimens were collected in Austria, Bosnia and Herzegovina, Bulgaria, France, Italy, Montenegro, Romania, and Spain.

### ﻿Whole genome sequencing and genome characterization of *R.lignumvallis* sp. nov.

Illumina sequencing resulted in 212,866,450 raw reads with a data amount of 31.9 Gbp for *R.lignumvallis* sp. nov. After trimming and contamination filtering, 173,132,236 reads (22.2 Gbp) were kept. The Genomescope2 analysis revealed a genome size of 699,853,381 bp and heterozygosity of 20% (see Suppl. material 2).

The NOVOplasty mitogenome assembly resulted in three contigs (18,087 bp, 1,404 bp, 238 bp) that could not be circularized. Therefore, the 15,623 bp long contig obtained by MitoZ was chosen for annotation. The annotation of the mitogenome revealed all expected 13 protein-coding genes and both rRNAs and 23 tRNAs.

The nuclear genome assembly of *R.lignumvallis* sp. nov. contains 206,802 scaffolds with a total length of 644 Mb, an N50 of 5.6 kb, and a GC of 30%. The BUSCO search with 2,124 Endopterygota orthologs resulted in 82.5% BUSCOs; of these, 47.9% were complete (47.4% single, 0.5% duplicated), and 34.6% were fragmented. 96.3% of the reads were mapped back to the original assembly. Blobtools detected no contamination in the assembly for *R.lignumvallis* sp. nov. (see Suppl. material 2). However, some contamination was detected by NCBI using the improved FCS-GX screen according to https://github.com/ncbi/fcs (see Suppl. material 2).

The genomic characterization of *R.tsurakiana* is included in Suppl. material 3.

### ﻿Species description

#### ﻿ Rhycophila lignumvallis

Taxon classificationAnimaliaTrichopteraRhyacophilidae

Graf & Rázuri-Gonzales sp. nov.

1717976C-21BB-506E-8E9F-B1D1BFE1B61E

https://zoobank.org/5A58EBFA-E945-4032-917A-877444F5DA2B

##### Holotype.

France • ♂; Corsica, bridge over the river Vecchio near the confluence with the river Tavignano; 42.2275°N, 9.24306°E; 195 m a.s.l.; 25 Jul. 2019; col. W. Graf leg.; in ethanol; SMF (SMFTRI00018634).

##### Diagnosis.

*Rhyacophilalignumvallis* sp. nov. (Figs 4, 5A, B) belongs to the *Rhyacophilatristis* species group (Schmid 1970). It is most similar to *R.pubescens* (Fig. 5C, D), *R.tsurakiana* (Fig. 5E, F), *R.ligurica* Oláh & Vinçon, 2021 (in Oláh et al. 2021, figs 55–57 therein), *R.harmasa* Oláh & Vinçon, 2021 (in Oláh et al. 2021, figs 52–54 therein), and *R.abruzzica* Oláh & Vinçon, 2021(in Oláh et al. 2021, figs 49–51 therein), but *R.lignumvallis* sp. nov. is distinguishable from these species by the shape of tergum X, the dorsal arm of the phallic apparatus in lateral and ventral views, the shape of the aedeagus and parameres, and the second segment of the inferior appendages.

The dorsal surface of segment X is convex in all these species but narrower and higher in the new species, *R.tsurakiana*, and *R.harmasa*. In dorsal view, however, the new species has a slightly membranous, mesally notched, and inflated segment X, while segment X in *R.tsurakiana* appears flatter. Additionally, the dorsal branch of tergum X is rounded and broader in the new species, while it is narrower in *R.tsurakiana* and *R.harmasa*.

The dorsal appendix of the phallic apparatus in the new species is longer than the aedeagus and the parameres (Fig. 4D). This also occurs in *R.harmasa*, *R.ligurica*, *R.pubescens*, and *R.tsurakiana* but not in *R.abruzzica*. However, the shape of the dorsal appendix in lateral view in the new species is digitate and slightly curved dorsad, whereas *R.harmasa* has a slightly wider apical half, *R.ligurica* has a low bump mesally on its dorsal surface, and *R.tsurakiana* has a straight and flat dorsal appendix. In *R.abruzzica*, the dorsal appendage is broad and medially widened in lateral view. In comparison to *R.lignumvallis* sp. nov. (Figs 4D, 5A, B), *R.tsurakiana*, and *R.abruzzica*, the dorsal appendix of the phallic apparatus is much longer and clearly exceeds segment X in dorsal view in *R.harmasa*, *R.pubescens*, and *R.ligurica*. In ventral view, the dorsal appendix is straight and rounded apically in the new species (Fig. 4E), slightly inflated on the apical half and rounded apically in *R.harmasa*, almost straight and truncate apically in *R.ligurica*, constricted basally and truncate apically in *R.tsurakiana*, and rectangular in *R.abruzzica*.

The aedeagus and parameres in *R.lignumvallis* sp. nov. are most similar to *R.pubescens*. However, in lateral view, the tip of the aedeagus in the new species is slenderer and slightly more curved apically than in *R.pubescens*. In lateral view, the parameres in the new species are broader than in *R.pubescens*. In ventral view, the parameres in the new species are club-shaped and curved mesad, while in *R.pubescens*, the parameres are digitate and directed posterad.

The second segment of the inferior appendages in the new species is triangular, with a straight dorsal margin, while all the other species have a concave dorsal margin (Fig. 4A, 5B).

##### Description.

**Figure 3.**
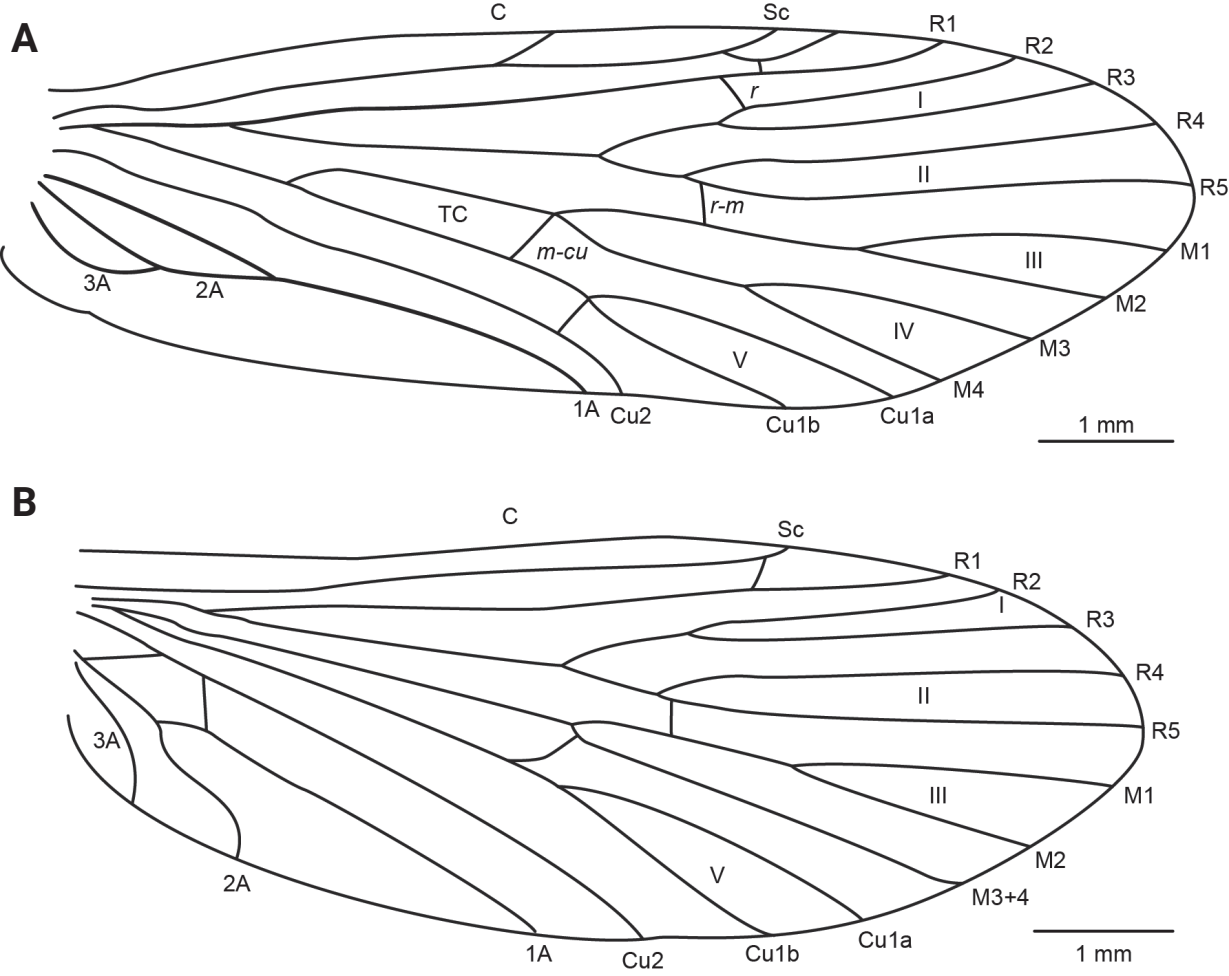
*Rhyacophilalignumvallis* sp. nov., wing venation. **C**, costal vein; **Sc**, subcostal vein; **R1–R5**, first to fifth branches of the radial vein; **M1–M4**, first to fourth branches of the medial vein; **M3+4**, medial vein 3+4 (hindwing); **Cu1a**, anterior branch of first cubital vein; **Cu1b**, posterior branch of first cubital vein; **Cu2**, second cubital vein; **1A–3A**, first to third anal veins; **r**, radial crossvein; **r-m**, radiomedial crossvein; **m-cu**, mediocubital crossvein; **I–V**, first to fifth wing forks; **TC**, thyridial cell. Scale bar: 1 mm. Illustrations were produced by Ernesto Rázuri-Gonzales.

***Adult male*.** Specimen in ethanol, mostly denuded; dorsally brown, ventrally light brown. Legs light brown with slightly darker tibial spurs. Head with frontal setal wart triangular; antennal setal wart subtriangular and smaller than frontal setal wart; posterior setal warts oval and connected to ocellar setal warts via a raised cuticular “bridge” (see Schmid, 1970; pl. I, fig. 1). Forewing length (8.8 mm, *N* = 1) mostly denuded, with sparse, very short light brown setae and golden brown microtrichia. Hind wings also mostly denuded, with slightly longer light brown setae. Forewing (Fig. 3A) with crossveins connecting costal (C) and subcostal (Sc) veins; subcostal (Sc) and first radial (R_1_) veins, first (R_1_) and second radial (R_2_) veins (*r*), fifth radial (R_5_) and first medial (M) veins (*r-m*), first medial (M) and first cubital (Cu1) veins (*m-cu*), and first cubital (Cu1) and second cubital (Cu2) veins. Hind wing (Fig. 3B) with crossveins connecting subcostal (Sc) and first radial (R_1_) veins, fifth radial (R_5_) and first medial (M) veins (*r-m*), and M_3+4_ and first cubital (Cu1) present.

**Figure 4.**
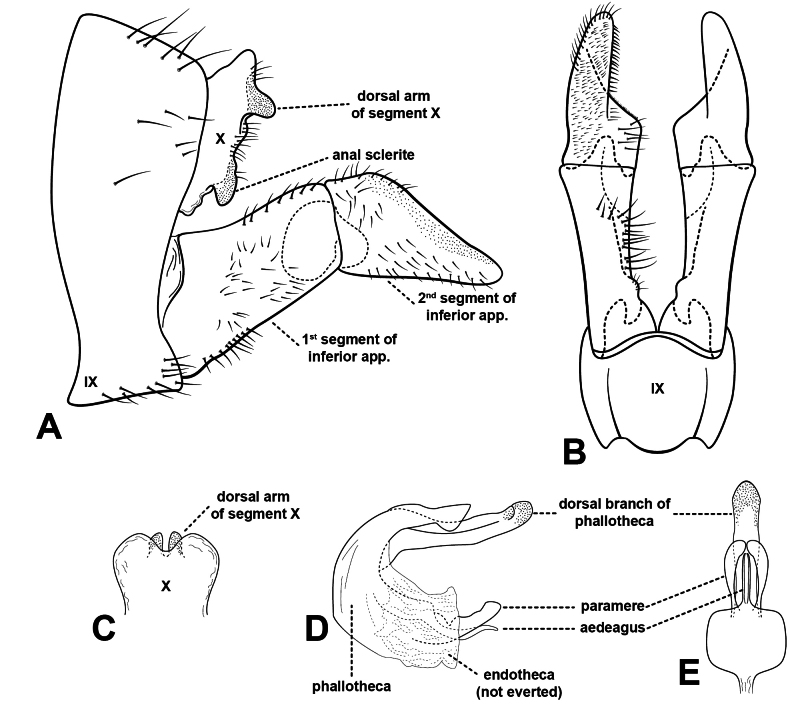
*Rhyacophilalignumvallis* sp. nov., male genitalia, lateral view (**A**), ventral view (**B**), segment X in dorsal view (**C**), phallic apparatus in lateral view (**D**), and phallic apparatus in ventral view (**E**). Illustrations were produced by Ernesto Rázuri-Gonzales.

***Male genitalia*.** Segment IX longitudinally short in lateral view (Fig. 4A), anterior and posterior margins slightly concave, dorsal half slightly longer than ventral. Dorsal surface of segment X membranous, slightly inflated, shallowly notched mesally in dorsal view (Fig. 4C). Dorsal branch of segment X short and rounded in lateral view. Anal sclerites partially fused to each other basally and to segment X, in lateral view, directed ventrad. First article of inferior appendages (Fig. 4A) rectangular in lateral view, slightly broader basally than apically; in ventral view (Fig. 4B), slightly broader apically than basally, with a small setose bump basally on mesal surface. Second article of inferior appendages (Fig. 4A) quadrangular in lateral view, dorsal and ventral margins slightly diverging, posterodorsal margin straight, at a 130° angle to dorsal margin; in ventral view (Fig. 4B), mitton-shaped, mesal margin with very short, peg-like setae basally and longer setae apically. Phallic apparatus (Fig. 4D) with dorsal appendix straight in lateral view, slightly curved dorsad, rounded apically, longer than parameres and aedeagus; in ventral view, straight, lateral margins slightly sinuous, rounded apically. Parameres in lateral view (Fig. 4D) broader than aedeagus, slightly curved posterodorsad, ventral margin straight, dorsal margin slightly sinuous, rounded apically; in ventral view (Fig. 4E), club-shaped, directed mesad. Aedeagus in lateral view (Fig. 4D) slender, slightly sinuous, tapering towards its apex; in ventral view, slender and straight.

**Figure 5.**
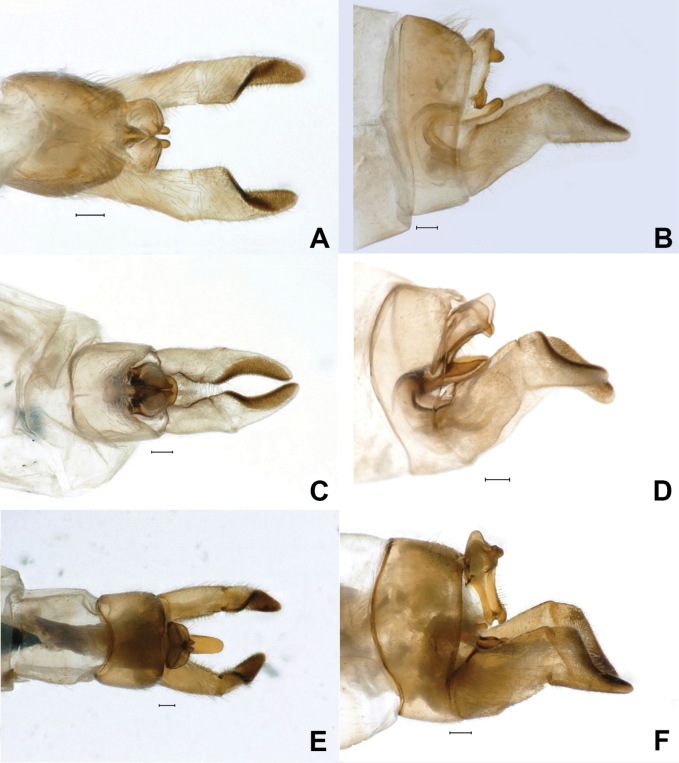
*Rhyacophilalignumvallis* sp. nov., male genitalia, dorsal view (**A**), lateral view (**B**). *R.pubescens*, male genitalia, dorsal view (**C**), lateral view (**D**). *R.tsurakiana*, male genitalia, dorsal view (**E**), lateral view (**F**). Scale bars: 100 μm. Specimens were photographed by W. Graf.

##### Etymology.

We dedicate this species to Dr Ralph W. Holzenthal to honor his contributions to caddisfly taxonomy and systematics. *Lignumvallis*, wood valley, is derived from the Latin translation of Ralph’s last name.

##### Habitat.

The river Vecchio is a crystal-clear, slow-flowing stream with a heterogeneous bottom substrate that varies from sandy patches to gravel to boulders. Stabile substrates were densely covered by *Agapetuscyrnensis* pupae. As many spring trickles enter the river on its left margin and the specimen was collected by sweeping the vegetation, the habitat of *R.lignumvallis* sp. nov. remains unknown.

## ﻿Discussion

Despite being one of the most well-known faunas in the world, on average more than 770 new animal species are described from Europe each year (Fontaine et al. 2012), with many taxonomic groups not yet reaching a plateau (e.g., Mateos et al. 2017). This trend is particularly pronounced among endemic species, with conservative estimates suggesting that up to one-fifth of endemic taxa from Europe have not yet been described (Essl et al. 2013). Many of these narrowly distributed species are characterized by inhabiting very small, isolated habitats and generally having small populations, making them especially vulnerable to environmental changes (Hering et al. 2009; Essl et al. 2013). This vulnerability is augmented in species occurring at higher elevations that may be subject to “summit trap effects” (Bálint et al. 2011; Domisch et al. 2011; Sauer et al. 2011; Taubmann et al. 2011). Discovering and potentially safeguarding these species prior to their extirpation should be a priority in conservation efforts.

In the case of caddisflies, southern Europe (e.g., Spain, Italy, and the Balkan Peninsula), mountainous regions (e.g., the Alps and the Pyrenees), and the Caucasus have been shown to be particularly species-rich and centers of endemism (Pauls et al. 2006; Previšić et al. 2014; Graf et al. 2015; Schmidt-Kloiber et al. 2017). Although caddisflies are well-studied in most of Europe, further studies in these highly diverse areas are necessary to better understand their richness in this continent and will likely yield many more new species.

The *Rhyacophilatristis* group now includes 29 species distributed throughout central-southern Europe and Western Asia, with many of them occurring in biodiversity centers in these regions (e.g., southern Europe and the Balkans, Suppl. material 1). Additionally, many species in this group are only known from a single locality, a single or very few specimens, or with unknown females/immature stages. For example, the single adult specimen of *R.lignumvallis* sp. nov. was associated with larval specimens from Corsica, previously identified as *R.pubescens* (Engelhardt 2009). Further sampling will clarify the potential presence of *R.pubescens* on the island. This suggests that the taxonomy of this group is far from complete, particularly for juvenile stages.

Aquatic insects have traditionally been neglected in genomic research (Hotaling et al. 2020). Using 40× coverage of short-read sequencing, we were able to obtain a draft nuclear and complete mitogenome assembly for the holotype of *R.lignumvallis* sp. nov. The assembly of the newly described species was 644,010,216 bp in length, which is close to the estimate obtained by Genomescope2. With an N50 of 5.6 Kbp, the genome assembly is less contiguous than previously published *Rhyacophila* genomes (*R.brunnea* Banks, 1911 and *R.evoluta* McLachlan, 1879 in Heckenhauer et al. (2023)). This lower contiguity is probably due to the sequencing technologies used (Oxford Nanopore long-reads followed by polishing with Illumina short-reads for the *R.brunnea* genome assembly vs. Illumina short-reads only for the *R.lignumvallis* sp. nov. genome) and/or sequencing coverage (97× and 116× Illumina filtered reads for the two *R.evoluta* genome assemblies vs. 40× in the new species) (Heckenhauer et al. 2022).

The percentage of BUSCOs recovered in the draft genome assembly was 82.5%. Of these, 47.9% were complete and 34.6% were fragmented. Meanwhile, the previously generated *Rhyacophila* genomes had a complete BUSCO score of 95.4% for *R.brunnea* (only 2.5% fragmented) and 74.1/75.1% for two *R.evoluta* specimens (17.9/18.7% fragmented) (Heckenhauer et al. 2022). The discrepancy in the amount of complete and fragmented BUSCOs among these congeners is probably due to the reasons outlined for the contiguity.

Genome assembly quality can be assessed using various metrics, such as contiguity and BUSCO completeness (Gurevich et al. 2013; Heckenhauer et al. 2022). Clearly, this genomic characterization is far from a reference genome quality, but nevertheless permanently connects the species name, the underlying morphology as preserved in the type specimen with the genetic make-up of the most representative specimen of the species (e.g., Hebert and Gregory 2005; Padial and de la Riva 2007; Pohl et al. 2012; Egan et al. 2017; Heckenhauer et al. 2023). This information is valuable for studying the systematics and evolution of the species in question as described in Heckenhauer et al. (2023). Additionally, the complete mitogenome includes the DNA barcode, which has already become important to species descriptions and can be used to monitor the occurrence of this species in freshwater bodies.

## Supplementary Material

XML Treatment for Rhycophila lignumvallis

## ﻿Additional information

### Author ORCIDs

Ernesto Rázuri-Gonzales https://orcid.org/0000-0002-7554-0816

Wolfram Graf https://orcid.org/0000-0001-6559-0644

Jacqueline Heckenhauer https://orcid.org/0000-0001-8771-9154

Julio V. Schneider https://orcid.org/0000-0002-9823-6569

Steffen U. Pauls https://orcid.org/0000-0002-6451-3425

### Data availability

All of the data that support the findings of this study are available in the main text or Supplementary Information.

## Supplementary materials

Supplementary material 1 Blobtools graphs for the *Rhyacophilalignumvallis* sp. nov. assembly Data type docx https://binary.pensoft.net/file/1180558This dataset is made available under the Open Database License (http://opendatacommons.org/licenses/odbl/1.0/). The Open Database License (ODbL) is a license agreement intended to allow users to freely share, modify, and use this Dataset while maintaining this same freedom for others, provided that the original source and author(s) are credited.Ernesto Rázuri-Gonzales, Wolfram Graf, Jacqueline Heckenhauer, Julio V. Schneider, Steffen U. Pauls

Supplementary material 2 Genomic methods and characterization for the *Rhyacophilatsurakiana* genome assembly Data type docx https://binary.pensoft.net/file/1180559This dataset is made available under the Open Database License (http://opendatacommons.org/licenses/odbl/1.0/). The Open Database License (ODbL) is a license agreement intended to allow users to freely share, modify, and use this Dataset while maintaining this same freedom for others, provided that the original source and author(s) are credited.Ernesto Rázuri-Gonzales, Wolfram Graf, Jacqueline Heckenhauer, Julio V. Schneider, Steffen U. Pauls

Supplementary material 3 Geographic distribution of species in the *Rhyacophilatristis* species group Data type docx Explanation note This list includes the geographic distribution of the 25 currently known species in the *Rhyacophilatristis* species group based on literature records. We only include papers with geographic data. https://binary.pensoft.net/file/1180560This dataset is made available under the Open Database License (http://opendatacommons.org/licenses/odbl/1.0/). The Open Database License (ODbL) is a license agreement intended to allow users to freely share, modify, and use this Dataset while maintaining this same freedom for others, provided that the original source and author(s) are credited.Ernesto Rázuri-Gonzales, Wolfram Graf, Jacqueline Heckenhauer, Julio V. Schneider, Steffen U. Pauls
