# Supplementary material for: ﻿A new species of Rhyacophila Pictet, 1834 (Trichoptera, Rhyacophilidae) from Corsica with the genomic characterization of the holotype
Source: Zookeys. 2024 Nov 22;1218:295–314. doi: 10.3897/zookeys.1218.132275 (PMC11607585; doi:10.3897/zookeys.1218.132275)
Supplement: Supplementary material 1 — Blobtools graphs for the Rhyacophilalignumvallis sp. nov. assembly [file zookeys-1218-295_article-132275__-s001.docx]

**SUPPLEMENTARY FILE 1**

**A new species of *Rhyacophila* Pictet 1834 (Trichoptera: Rhyacophilidae) from Corsica with the genomic characterization of the holotype**

Ernesto Rázuri-Gonzales, Wolfram Graf, Jacqueline Heckenhauer, Julio V. Schneider, Steffen U. Pauls

**Geographic distribution of species in the *Rhyacophila tristis* species group**

This list includes the geographic distribution of the 29 currently known species in the *Rhyacophila tristis* species group based on literature records. We only include papers with geographic data. The type country is indicated with an asterisk. Additionally, we include the species subgroup to which they belong.

***Rhyacophila aberrans* Martynov, 1913 [*tristis* subgroup]**

**Distribution:** Iran [?], Georgia*****, Turkey.

**References:** Martynov (1913); Sipahiler (2005; 2013).

***Rhyacophila abruzzica* Oláh & Vinçon, 2021 [*pubescens* subgroup]**

**Distribution:** Italy*****.

**References:** Oláh et al. (2021).

***Rhyacophila akutila* Oláh, 2010 [*tristis* subgroup]**

**Distribution:** Bulgaria*****.

**References:** Oláh (2010).

***Rhyacophila aquitanica* McLachlan, 1879 [*tristis* subgroup]**

**Distribution:** Austria, France*****, Germany, Italy, Romania, Switzerland.

**References:** Bálint et al. (2008); Bálint et al. (2009); Cianficconi et al. (2008); Coppa et al. (2012); McLachlan (1879); Oláh (2010); Ujvarosi et al. (2008).

***Rhyacophila borcka* Sipahiler, 1996 [*pubescens* subgroup]**

**Distribution:** Turkey*****.

**References:** Sipahiler (1996).

***Rhyacophila bosnica* Schmid, 1970 [*tristis* subgroup]**

**Distribution:** Bosnia and Herzegovina*****, Kosovo.

**References:** Ibrahimi et al. (2015); Ibrahimi et al. (2014); Ibrahimi et al. (2012b); Kalamujić Stroil et al. (2018); Musliu et al. (2020); Schmid (1970).

***Rhyacophila braaschi* Malicky & Kumanski, 1976 [*pubescens* subgroup?]**

**Distribution:** Bulgaria*****.

**References:** Kumanski and Malicky (1976).

***Rhyacophila carpathica* Botosaneanu, 1995 [*tristis* subgroup]**

**Distribution:** Romania*.

**References:** Bálint et al. (2009); Botosaneanu (1995).

***Rhyacophila cibinensis* Botosaneanu & Marinkovic, 1967 [*tristis* subgroup]**

**Distribution:** Romania*****.

**References:** Botosaneanu and Marinkovic (1967); Ujvarosi et al. (2008).

***Rhyacophila harmasa* Oláh & Vinçon, 2021 [*pubescens* subgroup]**

**Distribution:** Albania*****.

**References:** Oláh et al. (2021).

***Rhyacophila kozara* Oláh, 2022 [*tristis* subgroup]**

**Distribution:** Bosnia and Herzegovina*.

**References:** Oláh et al. (2022).

***Rhyacophila lignumvallis* sp. nov. [*pubescens* subgroup]**

**Distribution:** France*.

**References:** This manuscript.

***Rhyacophila ligurica* Oláh & Vinçon, 2021 [*pubescens* subgroup]**

**Distribution:** Italy*****.

**References:** Oláh et al. (2021).

***Rhyacophila margaritae* Kumanski, 1998 [*tristis* subgroup]**

**Distribution:** Bulgaria*****.

**References:** Kumanski (1998).

***Rhyacophila obtusa* Klapálek, 1894 [*tristis* subgroup]**

**Distribution:** Bulgaria*****, Greece, Kosovo, North Macedonia, Romania, Serbia, Turkey.

**References:** Ibrahimi et al. (2017); Ibrahimi et al. (2014); Ibrahimi et al. (2012b); Karaouzas and Malicky (2015); Klapálek (1894); Oláh (2010; 2011); Sipahiler (2016); Ujvarosi et al. (2008).

***Rhyacophila olahorum* Oláh, 2016 [*tristis* subgroup]**

**Distribution:** Romania*****.

**References:** Oláh and Beshkov (2016).

***Rhyacophila orghidani* Botosaneanu, 1952 [*tristis* subgroup]**

**Distribution:** Romania*****.

**References:** Botosaneanu (1952); Oláh and Balogh (2015); Oláh and Beshkov (2016); Ujvarosi et al. (2008).

***Rhyacophila pendayica* Malicky, 1975 [*tristis* subgroup]**

**Distribution:** Greece*****.

**References:** Malicky (1975); Oláh (2010).

***Rhyacophila pirinica* Kumanski, 1980 [*pubescens* subgroup?]**

**Distribution:** Bulgaria*****.

**References:** Kumanski (1980).

***Rhyacophila producta* McLachlan, 1879 [*pubescens* subgroup]**

**Distribution:** Austria*****, Italy.

**References:** Cianficconi et al. (2008); Lodovici and Valle (2020); McLachlan (1879); Mey (2023); Niedrist et al. (2017).

***Rhyacophila pseudotristis* Kumanski, 1987 [*tristis* subgroup]**

**Distribution:** Bulgaria*****.

**References:** Kumanski (1987).

***Rhyacophila pubescens* Pictet, 1834 [*pubescens* subgroup]**

**Distribution:** Austria, Czech Republic, France, Germany, Hungary, Italy, Luxembourg, North Macedonia, Poland, Slovakia, Switzerland*****.

**References:** Chvojka and Komzák (2008); Cianficconi et al. (2008); Cianficconi et al. (2005); Engelhardt et al. (2008); Kiss (2008); Mey (2023); Mosely (1930); Mosely (1932); Pictet (1834); Schrankel et al. (2008); Slavevska-Stamenković et al. (2020); Valle and Lodovici (2018).

***Rhyacophila sarplana* Oláh, 2022 [*tristis* subgroup]**

**Distribution:** Albania, North Macedonia*.

**References: Oláh (2010);** Oláh et al. (2022).

***Rhyacophila spinulata* Martynov, 1913 [*tristis* subgroup]**

**Distribution:** Georgia*****, Turkey.

**References:** Martynov (1913); Sipahiler (2005; 2013).

***Rhyacophila staraplana* Oláh, 2022 [*tristis* subgroup]**

**Distribution:** Serbia*.

**References:** Oláh et al. (2022).

***Rhyacophila trescavicensis* Botosaneanu, 1960 [*tristis* subgroup]**

**Distribution:** Albania, Bosnia and Herzegovina*****, Montenegro, Kosovo.

**References:** Botosaneanu (1960); Ibrahimi et al. (2014); Ibrahimi et al. (2012b); Oláh (2010; 2011).

***Rhyacophila tristis* Pictet, 1834 [*tristis* subgroup]**

**Distribution:** Albania, Andorra, Austria, Bulgaria, Croatia, Czech Republic, France, Germany, Greece, Hungary, Italy, Kosovo, Luxembourg, North Macedonia, Montenegro, Portugal, Romania, Serbia, Slovakia, Spain, Switzerland*****, Turkey, Ukraine.

**References:** Bálint et al. (2009); Bálint et al. (2011); Bonada et al. (2004); Céréghino (2002); Chvojka and Komzák (2008); Cianficconi et al. (2011); Cianficconi et al. (2008); Cianficconi et al. (2005); Coppa et al. (2012); Dohet et al. (2008); Gashi et al. (2015); González González and Martínez Menéndez (2011); Ibrahimi and Gashi (2008); Ibrahimi et al. (2015); Ibrahimi et al. (2017); Ibrahimi et al. (2012a; 2014); Ibrahimi et al. (2012b); Karaouzas and Malicky (2015); Kiss (2008); Krno (1990); Kruijt (2017); Kučinić et al. (2020); Kučinić et al. (2021); Malicky (2014); Martín et al. (2014); Martín et al. (2016); Martínez et al. (2015); Martínez et al. (2016); Martínez Menéndez and González (2009); Martínez Menéndez and González (2010); Martini and Waringer (2021); Mey (2023); Mosely (1930); Nowinszky et al. (2014); Oláh (2010); Oláh and Balogh (2015); Oláh and Kovács (2013); Pictet (1834); Schrankel et al. (2008); Sipahiler (2005; 2007; 2013; 2016); Szczesny and Godunko (2008); Ujvarosi et al. (2008); Valladolid et al. (2011); Valle and Lodovici (2018); Waringer et al. (2018); Wolf and Angersbach (2010)

***Rhyacophila tsurakiana* Malicky, 1984 [*pubescens* subgroup]**

**Distribution:** Albania, Greece*****.

**References:** (Malicky 1984; Oláh 2010; Oláh and Kovács 2013; Waringer et al. 2018).

***Rhyacophila vranitzensis* Botosaneanu & Marinkovic, 1967 [*tristis* subgroup]**

**Distribution:** Bosnia and Herzegovina*.

**References:** Botosaneanu and Marinkovic (1967); Oláh (2010).

**References**

Bálint M, Barnard PC, Schmitt T, Ujvarosi L, Popescu O (2008) Differentiation and speciation in mountain streams: a case study in the caddisfly *Rhyacophila aquitanica* (Trichoptera). *Journal of Zoological* Systematics and Evolutionary Research 46: 340-345

Bálint M, Botosaneanu L, Ujvarosi L, Popescu O (2009) Taxonomic revision of *Rhyacophila aquitanica* (Trichoptera: Rhyacophilidae), based on molecular and morphological evidence and change of taxon status of *Rhyacophila aquitanica* ssp. *carpathica* to *Rhyacophila carpathica* stat. n. Zootaxa 2148: 39-48

Bálint M, Ujvarosi L, Denes AL, Popescu O (2011) European phylogeography of *Rhyacophila* *tristis* Pictet (Trichoptera: Rhyacophilidae): preliminary results. Zoosymposia 5: 11-18

Bonada N, Zamora-Muñoz C, Rieradevall M, Prat N (2004) Trichoptera (Insecta) collected in Mediterranean river basins of the Iberian Peninsula: taxonomic remarks and notes on ecology. Graellsia 60: 41-69

Botosaneanu L (1952) *Rhyacophila orghidani* n. sp. (Trichoptera Rhyacophilinae) din Muntii Apuseni ai Republicii Populare Române. Comunicările Academiei Republicii Populare Române 2: 721-724

Botosaneanu L (1960) Trichoptères de Yougoslavie recueillis en 1955 par le Dr. F. Schmid. Deutsche Entomologische Zeitschrift 7: 261-293

Botosaneanu L (1995) Additional documents to the knowledge of the Trichoptera of Romania, with data on European taxa from outside this country (Insecta: Trichoptera). Faunistische Abhandlungen Staatliches Museum für Tierkunde Dresden 20: 57-88

Botosaneanu L, Marinkovic M (1967) Sur quelques *Rhyacophila* du groupe de *tristis* (Trichoptera). Annales de la Société Entomologique de France (NS) 3: 1145-1151

Céréghino R (2002) Shift from a herbivorous to a carnivorous diet during the larval development of some *Rhyacophila* species (Trichoptera). Aquatic Insects 24: 129-135

Chvojka P, Komzák P (2008) The history and present state of Trichoptera research in the Czech Republic. Ferrantia 55: 11-21

Cianficconi F, Corallini C, La Porta G, Todini B (2011) Trichopteran fauna in a region of Central Italy: Lazio. Zoosymposia 5: 41-62

Cianficconi F, Corallini C, Todini B (2008) The genus *Rhyacophila* Pictet, 1834 in Italy. Ferrantia 55: 22-32

Cianficconi F, Todini B, Pedrotti CC (2005) Italian caddisflies living on mosses: a preliminary note. In: Tanida K, Rossiter A (Eds) Proceedings of the 11th International Symposium on Trichoptera. Tokai University Press, Kanagawa, 91-99

Coppa G, Graf W, Tachet H (2012) A revised description of the larvae of three species of the *Rhyacophila tristis* group: *Rhyacophila aquitanica*, *Rhyacophila pubescens* and *Rhyacophila tristis* (Trichoptera: Rhyacophilidae). Annales de Limnologie 48: 215-223. doi: <https://doi.org/10.1051/limn/2012014>

Dohet A, Ferreol M, Cauchie H-M, Hoffmann L (2008) Caddisfly assemblages characterizing different ecological areas in Luxembourg: from geographical distributions to bioindication. Ferrantia 55: 33-56

Engelhardt CHM, Pauls SU, Haase P (2008) Population genetic structure of the caddisfly *Rhyacophila pubescens*, Pictet 1834, north of the Alps. Fundamental and Applied Limnology 173: 165-176

Gashi A, Ibrahimi H, Grapci-Kotori L, Sejdiu N, Bislimi K (2015) New records of *Drusus siveci* Malicky, 1981 (Trichoptera, Limnephilidae, Drusinae) from the Balkan Peninsula, with ecological notes. Acta Zoologica Bulgarica 67: 259-264

González González MA, Martínez Menéndez J (2011) Checklist of the caddisflies of the Iberian Peninsula and Balearic Islands (Trichoptera). Zoosymposia 5: 115-135

Ibrahimi H, Gashi A (2008) State of knowledge of investigations on Trichoptera larvae in Kosova. Ferrantia 55: 70-72

Ibrahimi H, Gashi A, Kotori LG, Etemi FZ, Bilalli A, Musliu M (2015) New Distribution and Species Records of Caddisflies (Insecta: Trichoptera) from the Republic of Kosovo. Entomological News 125: 229-238

Ibrahimi H, Jahiji E, Bilalli A (2017) New records for the caddisfly (Insecta: Trichoptera) fauna of Serbia. Entomological News 127: 185-191

Ibrahimi H, Kucinic M, Gashi A, Grapci-Kotori L (2012a) The caddisfly fauna (Insecta, Trichoptera) of the rivers of the Black Sea basin in Kosovo with distributional data for some rare species. ZooKeys 182: 71-85

Ibrahimi H, Kucinic M, Gashi A, Grapci-Kotori L (2014) Trichoptera biodiversity of the Aegean and Adriatic Sea basins in the Republic of Kosovo. Journal of Insect Science 14. doi: <https://doi.org/10.1093/jisesa/ieu071>

Ibrahimi H, Kucinic M, Gashi A, Grapci-Kotori L, Vuckovic I, Cerjanec D (2012b) The genus *Rhyacophila* Pictet, 1834 (Insecta: Trichoptera) in Kosovo. Aquatic Insects 34: 23-31. doi: <https://doi.org/10.1080/01650424.2012.643021>

Kalamujić Stroil B, Lasić L, Hanjalić J, Mačar S, Vesnić A (2018) The first DNA barcode record for *Rhyacophila bosnica* Schmid, 1970 and pairing of adult and larval life stages. Genetics & Applications 2: 20-27. doi: <https://doi.org/10.31383/ga.vol2iss2pp20-27>

Karaouzas I, Malicky H (2015) New faunistic records of Trichoptera in Greece. Braueria 42: 13-20

Kiss O (2008) The Trichoptera (Insecta) of the Ban Stream, Bukk Mts., northern Hungary. Ferrantia 55: 73-79

Klapálek F (1894) Descriptions of new species of *Raphidia*, L., and of three new species of Trichoptera from the Balkan Peninsula, with critical remarks on *Panorpa gibberosa*, McLachlan. Transactions of the Entomological Society of London 1894: 489, 495

Krno Ij (1990) Longitudinal changes in the structure of macrozoobenthos and its microdistribution in natural and moderately eutrophicated waters of the River Rajčianka (Strážovské vrchy). Acta Facultatis Rerum Naturalium Universitatis Comenianae Zoologia 33: 31-48

Kruijt DB (2017) Waarnemingen van schietmotten in Luxemburg in 2014. De Digitale Kokerjuffer 20: 14-19

Kučinić M, Ćukušić A, Žalac S, Delić A, Cerjanec D, Podnar M, Ćuk R, Vučković I, Previšić A, Vuković M, Stanić Koštroman S, Bukvić V, Šalinović A, Plantak M (2020) Springs: DNA barcoding of caddisflies (Insecta, Trichoptera) in Croatia with notes on taxonomy and conservation biology. Natura Croatica 29: 73-98. doi: <https://doi.org/10.20302/NC.2020.29.8>

Kučinić M, Šalinović-Steinbacher A, Žalac S, Gumhalter D, Hlebec D, Cukušić A, Vučković I, Šašić M, Mihoci I, Hađina J, Vajdić M (2021) Faunal features of caddisflies (Insecta, Trichoptera) in Konavle region (Croatia) with notes on DNA barcoding and conservation biology. Natura Croatica 30: 331-350. doi: <https://doi.org/10.20302/NC.2021.30.21>

Kumanski KP (1980) Description of three new caddis-flies (Trichoptera) from Bulgaria. Rivista di Idrobiologia 19: 197-206

Kumanski KP (1987) On the group of *tristis* of genus *Rhyacophila* Pictet in Bulgaria with description of a new species (Trichoptera, Rhyacophilidae). Acta Zoologica Bulgarica 35: 16-22

Kumanski KP (1998) *Rhyacophila margaritae* - a new insect species (Trichoptera: Rhyacophilidae) from Bulgaria. Dokladi na Bulgarskata Akademiya na Naukite 51: 59-62

Kumanski KP, Malicky H (1976) Beiträge zur Kenntnis der bulgarischen köcherfliegen (Trichoptera). Polskie Pismo Entomologiczne 46: 95-126

Lodovici O, Valle M (2020) Nuovi dati sui Tricotteri Italiani (Insecta, Trichoptera): Rhyacophilidae, Glossosomatidae, Hydroptilidae e Philopotamidae. Rivista del Museo Civico di Scienze Naturali “Enrico Caffi”, Bergamo 33: 71-81

Malicky H (1975) Fünfzehn neue mediterrane köcherfliegen. Mitteilungen der Entomologischen Gesellschaft Basel 25: 81-96

Malicky H (1984) Fünf neue griechische Köcherfliegen (Trichoptera). Mitteilungen der Entomologischen Gesellschaft Basel 34: 96-102

Malicky H (2014) Mißgebildete Köcherfliegen (Trichoptera). Braueria 41: 5-31

Martín L, Martínez J, González MA (2014) Observaciones sobre los tricópteros (Insecta: Trichoptera) de las montañas orientales de Galicia (Sierras de Ancares, Courel e Invernadeiro). Boletín de la Asociación Española de Entomología 38: 67-90

Martín L, Martínez J, González R, González MA (2016) Caddisflies (Insecta, Trichoptera) from Montana Palentina (Parque Natural de las Fuentes Carrionas y Fuente Cobre) and Sierra de la Cabrera (Leon). Boletín de la Asociación Española de Entomología 40: 251-268

Martínez J, Martín L, González MA (2015) Tricópteros (Insecta: Trichoptera) de la serra do Xistral (Galicia, NO de España). Nova Acta Científica Compostelana (Bioloxía) 22: 33-47

Martínez J, Martín L, González MA (2016) New data on the caddisflies (Insecta, Trichoptera) of Asturias (N. Spain). Boletín de la Asociación Española de Entomología 40: 43-66

Martínez Menéndez J, González MA (2009) Observaciones sobre los Tricópteros de la Península Ibérica. XI: Tricópteros de Cataluña (NE de España) (Insecta: Trichoptera). Boletín de la Asociación Española de Entomología 33: 337-353

Martínez Menéndez J, González MA (2010) Notes on the caddisflies of the Iberian Peninsula, XII. The caddisflies of Andorra (NE Iberian Peninsula) (Insecta: Trichoptera). Boletín de la Asociación Española de Entomología 34: 113-121

Martini J, Waringer J (2021) Dynamic microhabitat shifts in space and time of caddisfly larvae (Insecta: Trichoptera) in a first‐order calcareous mountain stream. Biologia 76: 2527-2541. doi: <https://doi.org/10.1007/s11756-021-00741-w>

Martynov AV (1913) Contributions a la faune des Trichoptères du Caucase. II. Trichoptères de la province de batoum et des environs du Novyj Afon. Horae Societatis Entomologicae Rossicae 40: 30.

McLachlan R (1879) A monographic revision and synopsis of the Trichoptera of the European fauna. Part 8. John van Voorst, London, 429-500, plates 445-451 pp.

Mey W (2023) Contribution to the caddisfly fauna of the Nature Park "Alpi Marittime" in Italy with a note on the endemic Trichoptera species of the Alps (Insecta, Trichoptera). Lauterbornia 89: 239-262

Mosely ME (1930) Corsican Trichoptera. Eos – Revista Española de Entomología 6: 147-184

Mosely ME (1932) Corsican Trichoptera and Neuroptera (s. l.). Eos – Revista Española de Entomología 8: 165-184

Musliu M, Ibrahimi H, Bilalli A, Stamenković VS, Hinić J, Bozdoğan H (2020) New Records for the Caddisfly Fauna (Insecta: Trichoptera) of the Karadak Mountains, Western Balkans. Journal of the Entomological Research Society 22: 153-162

Niedrist GH, Alber R, Scotti A, Rauch H, Vorhauser S, Kiebacher T, Bottarin R (2017) Aquatic invertebrates along the progression of glacial and non-glacial streams in Matsch Valley (South Tyrol, Italy). Gredleriana 17: 129-140

Nowinszky L, Kiss O, Puskas J (2014) Swarming patterns of light trapped individuals of caddisfly species (Trichoptera) in Central Europe. Central European Journal of Biology 9: 417-430

Oláh J (2010) New species and new records of Palaearctic Trichoptera in the material of the Hungarian Natural History Museum. Annales Historico-Naturales Musei Nationalis Hungarici 102: 65-117

Oláh J (2011) New species and records of Balkan Trichoptera. Folia Historico Naturalia Musei Matraensis 35: 111-121

Oláh J, Balogh C (2015) New records of the Carpathian Trichoptera. Folia Historico-Naturalia Musei Matraensis 39: 85-97

Oláh J, Beshkov S (2016) New records of Trichoptera in the Balkan Peninsula and Romania, with description of new *Rhyacophila* sibling species by speciation traits. Folia Entomologica Hungarica 77: 87-104

Oláh J, Kovács T (2013) New species and records of Balkan Trichoptera II. Folia Historico Naturalia Musei Matraensis 37: 109-121

Oláh J, Vinçon G, Coppa G (2021) On the Trichoptera of Italy with delineation of incipient sibling species. Opuscula Zoologica (Budapest) 52: 03-67. doi: <https://doi.org/10.18348/opzool.2021.1.3>

Oláh J, Beshkov S, Ibrahimi H, Kovács T, Oláh Jr. J, Vinçon G (2022) On the Trichoptera of the Balkan: survey on species complexes of *Polycentropus iarapetra*, *Rhyacophila balcanica*, *R*. *bosnica* and *Notidobia nekibe*. Opuscula Zoologica (Budapest) 53: 67-111. doi: https://doi.org/10.18348/opzool.2022.1.67

Pictet FJ (1834) Recherches pour servir à l'histoire et l'anatomie des Phryganides. A. Cherbuliez, Geneva, plates 1-20 + 235, 220 plates pp.

Schmid F (1970) Le genre *Rhyacophila* et la famille des Rhyacophilidae (Trichoptera). Memoires de la Société Entomologique du Canada 66: 1-230

Schrankel I, Neu P, Dohet A, Schoos F (2008) Checklist of the Trichoptera of the Grand Duchy of Luxembourg - first revision. Ferrantia 55: 89-92

Sipahiler F (1996) Four new Trichoptera species from northern Anatolia (Rhyacophilidae, Glossosomatidae, Sericostomatidae). Opuscula Zoologica Fluminensia 149: 1-9

Sipahiler F (2005) A checklist of the caddisflies of Turkey (Trichoptera). In: Tanida K, Rossiter A (Eds) Proceedings of the 11th International Symposium on Trichoptera. Tokai University Press, Kanagawa, 393-405

Sipahiler F (2007) The Trichoptera fauna of north-western Turkey with the descriptions of a new species and of some previously unknown females (Philopotamidae, Sericostomatidae). Braueria 34: 36-42

Sipahiler F (2013) Revision of the *Rhyacophila stigmatica* Species Group in Turkey with descriptions of three new species (Trichoptera, Rhyacophilidae). Zootaxa 3669: 43-55

Sipahiler F (2016) Faunistic studies on the Trichoptera fauna of northwestern Turkey and Thrace. Braueria 43: 11-16

Slavevska-Stamenković V, Hinić J, Karaouzas I, Ibrahimi H, Mitić-Kopanja D, Bilalli A (2020) First record of *Rhyacophila pubescens* Pictet, 1834 (Trichoptera: Rhyacophilidae) in the Republic of North Macedonia with notes on its ecology and distribution. Ecologica Montenegrina 31: 28-34. doi: <https://doi.org/10.37828/em.2020.31.6>

Szczesny B, Godunko R (2008) Checklist of Ukrainian Trichoptera. Braueria 35: 11-20

Ujvarosi L, Robert SC, Neu P, Robert B (2008) First revision of the Romanian caddisflies (Insecta: Trichoptera). Part 1: systematic checklist (updated 12/2005). Ferrantia 55: 110-124

Valladolid M, Martinez-Bastida JJ, Arauzo M (2011) The Trichoptera fauna of the Oja River (La Rioja, Spain). Zoosymposia 5: 497-507

Valle M, Lodovici O (2018) I Tricotteri di Calabria (Insecta, Trichoptera). Rivista del Museo Civico di Scienze Naturali “Enrico Caffi”, Bergamo 31: 139-186

Waringer J, Karaouzas I, Malicky H (2018) The larvae of *Rhyacophila tsurakiana* Malicky 1984, *Rhyacophila gudrunae* Malicky 1972, and *Rhyacophila biegelmeieri* Malicky 1984, including an update for the larval key to the Greek species of genus *Rhyacophila* Stephens 1836 (Rhyacophilidae, Trichoptera). Zootaxa 4508: 85-100. doi: <https://doi.org/10.11646/zootaxa.4508.1.5>

Wolf B, Angersbach R (2010) Does an increase in mean annual temperature influence the occurrence of Plecoptera and Trichoptera species in a German upland stream? Lauterbornia 71: 135-146
