# Supplementary material for: ﻿A new species of Rhyacophila Pictet, 1834 (Trichoptera, Rhyacophilidae) from Corsica with the genomic characterization of the holotype
Source: Zookeys. 2024 Nov 22;1218:295–314. doi: 10.3897/zookeys.1218.132275 (PMC11607585; doi:10.3897/zookeys.1218.132275)
Supplement: Supplementary material 2 — Genomic methods and characterization for the Rhyacophilatsurakiana genome assembly [file zookeys-1218-295_article-132275__-s002.docx]

**SUPPLEMENTARY FILE 2. Genomic characterization of the holotype of *Rhyacophila lignumvallis* sp. nov.**

**A new species of *Rhyacophila* Pictet 1834 (Trichoptera: Rhyacophilidae) from Corsica with the genomic characterization of the holotype**

Ernesto Rázuri-Gonzales, Wolfram Graf, Jacqueline Heckenhauer, Julio V. Schneider, Steffen U. Pauls

**
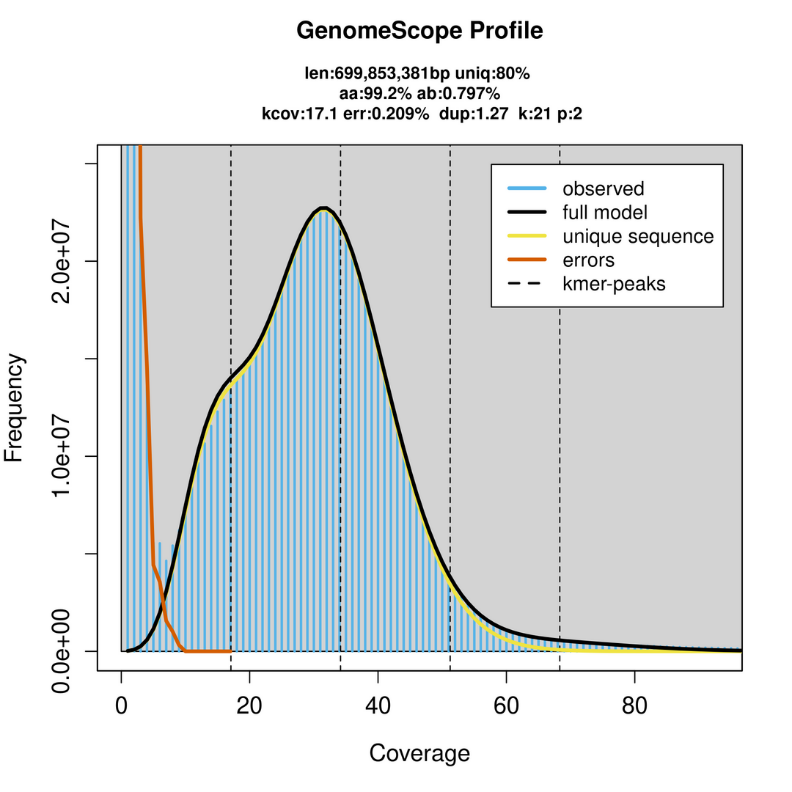
**

**Supplementary Figure 2.1.** GenomeScope profile for the holotype of *R*. *lignumvallis* **sp. nov.**, generated by GenomeScope2.

**
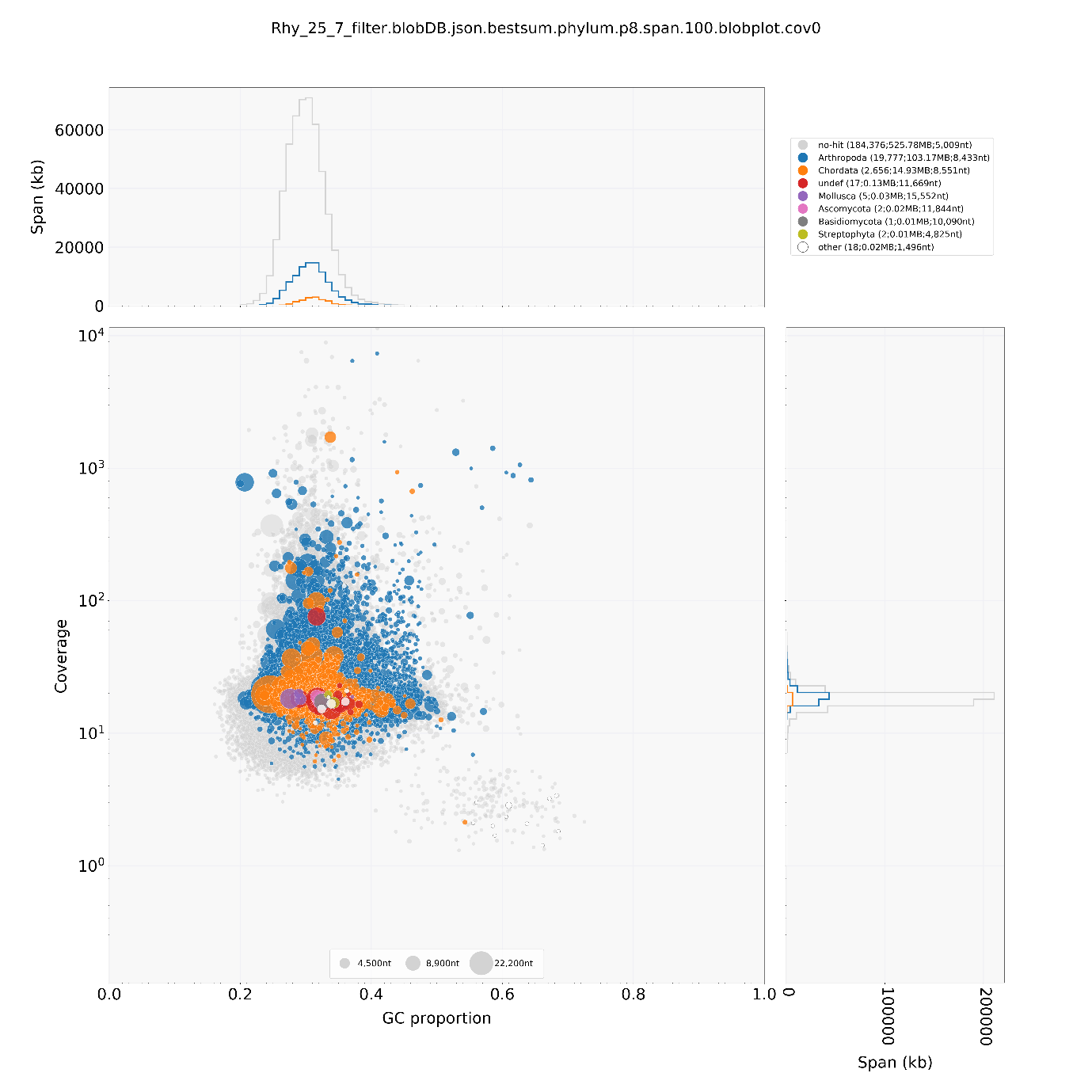
**

**Supplementary Figure 2.2.** Taxon-annotated GC-coverage (TAGC) plots for the nuclear genome assembly of *Rhyacophila lignumvallis* **sp. nov.** Scaffolds are represented with circles. Colors indicate the best match to the corresponding taxonomic annotation. The distribution of the total span (kb) of contigs for a given GC proportion or coverage is given in the upper- and right panels, respectively. NCBI removed the following contaminations using FCS-GX screen:

NODE_112825_length_1496_cov_2.430951 1496 prok:b-proteobacteria

NODE_149729_length_929_cov_16.360412 929 virs:viruses

NODE_153475_length_892_cov_3.113501 892 prok:b-proteobacteria

NODE_155354_length_874_cov_4.007326 874 prok:b-proteobacteria

NODE_156642_length_862_cov_1.701363 862 prok:b-proteobacteria

NODE_162481_length_807_cov_2.601064 807 prok:b-proteobacteria

NODE_166105_length_775_cov_2.256944 775 prok:b-proteobacteria

NODE_168434_length_755_cov_1.508571 755 prok:b-proteobacteria

NODE_169049_length_750_cov_2.189928 750 prok:b-proteobacteria

NODE_170444_length_738_cov_2.471449 738 prok:b-proteobacteria

NODE_180736_length_657_cov_2.677741 657 prok:b-proteobacteria

NODE_183491_length_637_cov_2.683849 637 prok:b-proteobacteria

NODE_183492_length_637_cov_1.647766 637 prok:b-proteobacteria

NODE_183772_length_635_cov_1.751724 635 prok:b-proteobacteria

NODE_183901_length_634_cov_2.972366 634 prok:b-proteobacteria

NODE_187178_length_612_cov_2.323160 612 prok:b-proteobacteria

NODE_187473_length_610_cov_1.821622 610 prok:b-proteobacteria

NODE_188824_length_601_cov_1.990842 601 prok:b-proteobacteria

NODE_189858_length_594_cov_2.803340 594 prok:b-proteobacteria

NODE_190975_length_587_cov_2.500000 587 prok:b-proteobacteria

NODE_192373_length_578_cov_2.615679 578 prok:b-proteobacteria

NODE_193066_length_574_cov_3.664740 574 prok:b-proteobacteria

NODE_194404_length_566_cov_2.432485 566 prok:b-proteobacteria

NODE_194882_length_563_cov_3.031496 563 prok:b-proteobacteria

NODE_194883_length_563_cov_2.029528 563 prok:b-proteobacteria

NODE_195379_length_560_cov_1.986139 560 prok:b-proteobacteria

NODE_196748_length_552_cov_2.215292 552 prok:b-proteobacteria

NODE_199273_length_538_cov_3.008282 538 prok:b-proteobacteria

NODE_200240_length_533_cov_3.872385 533 prok:b-proteobacteria

NODE_200848_length_530_cov_2.328421 530 prok:b-proteobacteria

NODE_201835_length_525_cov_2.887234 525 prok:b-proteobacteria

NODE_201837_length_525_cov_2.014894 525 prok:b-proteobacteria

NODE_202419_length_522_cov_2.049251 522 prok:b-proteobacteria

NODE_203022_length_519_cov_2.015086 519 prok:b-proteobacteria

NODE_204024_length_514_cov_2.847495 514 prok:b-proteobacteria

NODE_204025_length_514_cov_1.126362 514 prok:b-proteobacteria

NODE_204243_length_513_cov_2.465066 513 prok:b-proteobacteria

NODE_204656_length_511_cov_3.241228 511 prok:b-proteobacteria

NODE_204849_length_510_cov_1.828571 510 prok:b-proteobacteria

NODE_205844_length_505_cov_4.206667 505 prok:b-proteobacteria

NODE_206247_length_503_cov_4.013393 503 prok:b-proteobacteria

NODE_75828_length_2621_cov_21.890101 2621 anml:reptiles

NODE_81111_length_2411_cov_21.719015 2411 anml:fishes
