# Supplementary material for: ﻿A new species of Rhyacophila Pictet, 1834 (Trichoptera, Rhyacophilidae) from Corsica with the genomic characterization of the holotype
Source: Zookeys. 2024 Nov 22;1218:295–314. doi: 10.3897/zookeys.1218.132275 (PMC11607585; doi:10.3897/zookeys.1218.132275)
Supplement: Supplementary material 3 — Geographic distribution of species in the Rhyacophilatristis species group [file zookeys-1218-295_article-132275__-s003.docx]

**SUPPLEMENTARY FILE 3. Genomic methods and characterization of *Rhyacophila tsurakiana* genome assembly**

**A new species of *Rhyacophila* Pictet 1834 (Trichoptera: Rhyacophilidae) from Corsica with the genomic characterization of the holotype**

Ernesto Rázuri-Gonzales, Wolfram Graf, Jacqueline Heckenhauer, Julio V. Schneider, Steffen U. Pauls

## **Material and methods**

The specimen of *R*. *tsurakiana* Malicky, 1984 was collected in Albania, river Shushica at the village of Brataj (40°15'58.04"N, 19°40'19.13"E).

### *DNA extraction, library preparation, and whole genome sequencing*

The genomic methods were identical to the ones used for the holotype of *R*. *lignumvallis* **sp. nov.** Genomic libraries were prepared from 30.4 ng of sheared gDNA. The raw reads are deposited at the NCBI SRA archive under accession number SRR22799048 under Bioproject PRJNA899095.

## *Mitogenome and nuclear genome assembly*

The assembly for *R*. *tsurakiana* was identical to the ones for *R*. *lignuvallis* **sp. nov.**, and the mitochondrial genome assemblies were deposited in GenBank under the accession OQ984044 while the draft nuclear genome assembly was deposited in GenBank under accession JAPMAG000000000.

## **Results**

### *Whole genome sequencing and genome characterization of R. tsurakiana*

Illumina sequencing resulted in 204,684,090 raw reads with a data amount of 30.7 Gbp. After trimming and contamination filtering, 184,609,826 reads (21.8 Gbp) were kept for *R*. *tsurakiana*. The genome size of *R*. *tsurakiana* was estimated to be 782,328,465 bp, and heterozygosity was 27.6% (Supplemental Figure 3.1.)


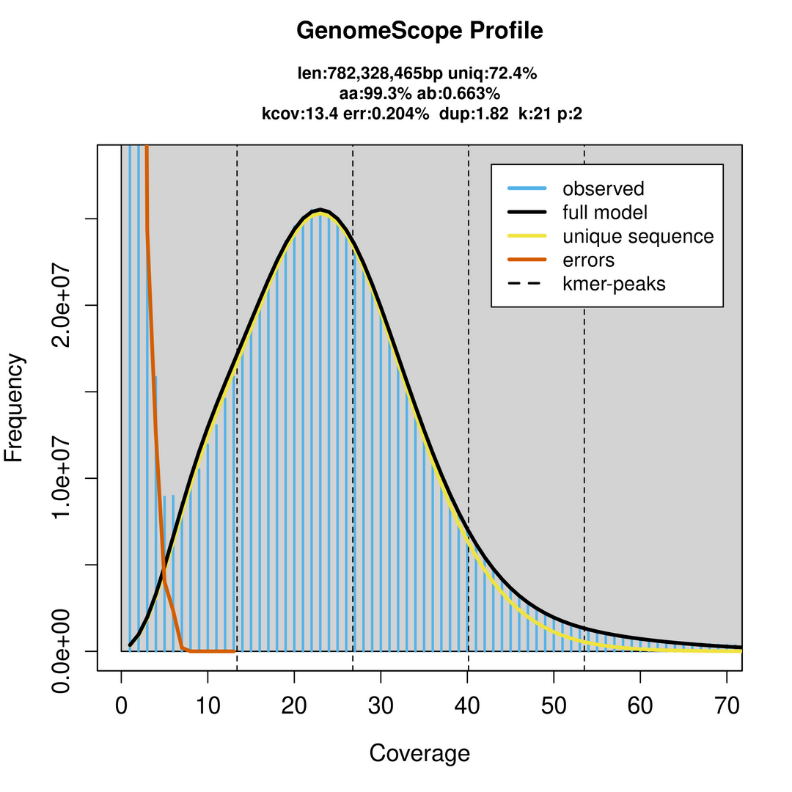


**Supplemental Figure 3.1.** GenomeScope profile for *R*. *tsurakiana*, generated by GenomeScope2.

While the MitoZ assembly resulted in one 15,340 bp, non-circular contig for *R*. *tsurakiana*, NOVOplasty assembled a slightly longer (total 15,877 bp) but otherwise identical, circular contig and was thus chosen for annotation. The annotation of the mitogenome of *R*. *tsurakiana* also revealed all expected 13 protein-coding genes and both rRNAs and 23 tRNAs.

The nuclear genome assembly of *R*. *tsurakiana* contains 283,758 scaffolds with a total length of 710.6 Mb, an N50 of 3.6 kb, and a GC of 31%. The BUSCO search with 2,124 Endopterygota orthologs resulted in 75.5% BUSCOs; of these, 35.1% were complete (34.7% single, 0.4% duplicated), and 40.4% were fragmented. 97.8% of the reads were mapped back to the *R*. *tsurakiana* assembly. While Blobtools detected no contaminations in the assemblies (Supplementary Figure 3.2.), NCBI's contamination screening detected and filtered out a 27bp and a 28bp long contamination (adaptor: NGB01088.1) at the beginning of two scaffolds in the nuclear assembly of *R*. *tsurakiana*.

**
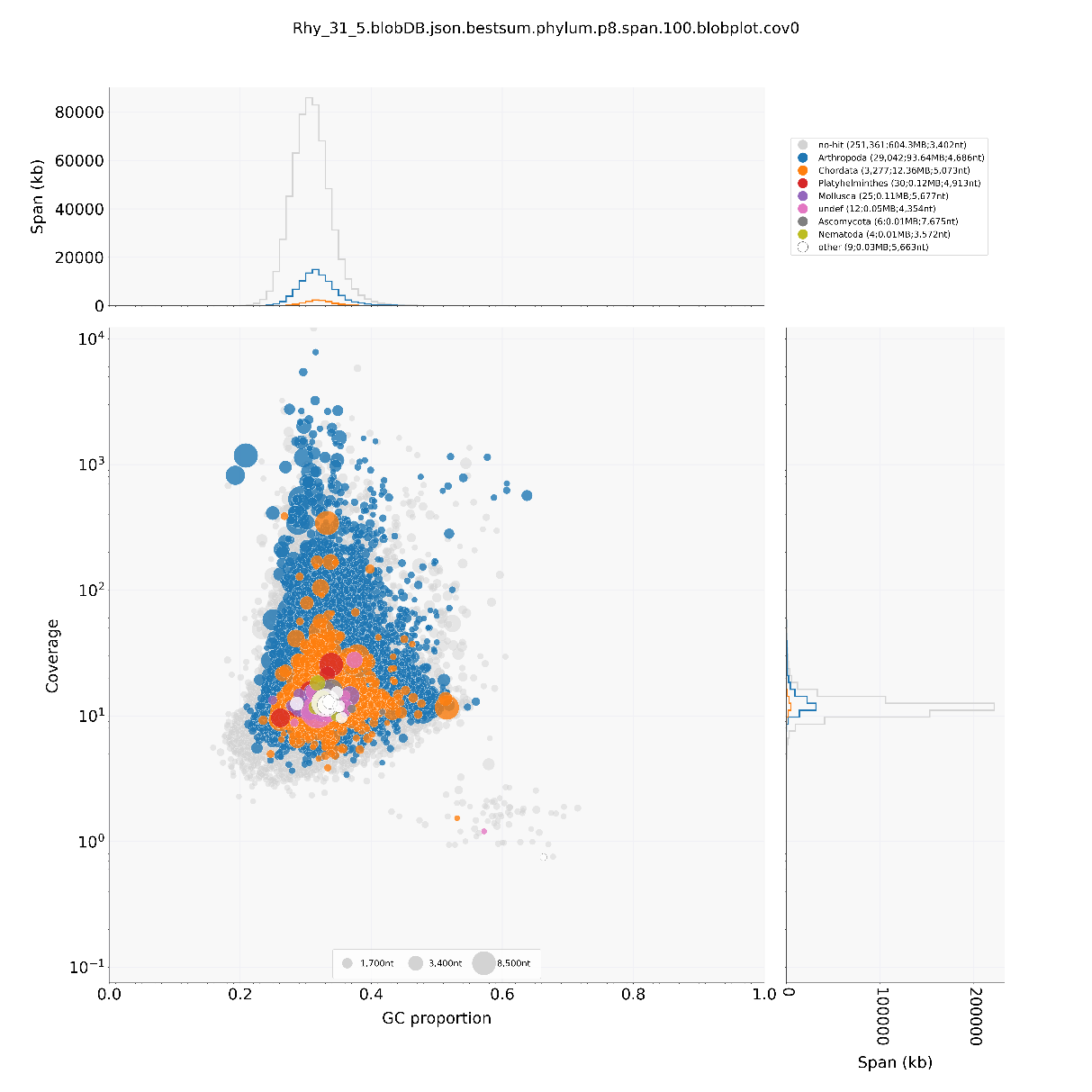
**

**Supplementary Figure 3.2.** Taxon-annotated GC-coverage (TAGC) plots for the nuclear genome assembly of *Rhyacophila tsurakiana*. Scaffolds are represented with circles. Colors indicate the best match to the corresponding taxonomic annotation. The distribution of the total span (kb) of contigs for a given GC proportion or coverage is given in the upper- and right panels, respectively. In addition, the new improved FCS-GX contamination screening of NCBI detected and excluded the following contaminations:

NODE_244039_length_773_cov_19.136490 773 anml:fishes

NODE_253223_length_707_cov_18.713190 707 anml:fishes

NODE_256355_length_686_cov_13.091918 686 anml:amphibians

NODE_258318_length_672_cov_17.072934 672 anml:fishes

NODE_259807_length_662_cov_13.546952 662 anml:fishes

NODE_280383_length_521_cov_12.306867 521 anml:fishes

NODE_283246_length_503_cov_1.504464 503 prok:b-proteobacteria
